# Satisfaction with Appearance and the Desired Treatment to Improve Aesthetics

**DOI:** 10.1155/2013/912368

**Published:** 2013-02-20

**Authors:** Bader K. Al-Zarea

**Affiliations:** Faculty of Dentistry, Al-Jouf University, P.O. Box 2232, Sakaka 42421, Saudi Arabia

## Abstract

*Objective*. To identify participants' satisfaction with appearance and the desired treatment to improve aesthetics. *Materials and Methods*. 220 participants (127 males and 93 females, mean age = 21.4 ± 1.5 years) were recruited into the study. A structured questionnaire was used to assess patients' satisfaction with appearance and what treatment they desire to improve aesthetics. Participants scored the level of satisfaction with appearance using visual analogue scale. *Results*. The VAS mean score of satisfaction with general appearance was 6.8 ± 2.3. Half participants were dissatisfied with tooth appearance and 65.9% were dissatisfied with tooth colour. Higher VAS scores were associated with higher desire for all treatments that improve tooth appearance (*P* < .05). Dissatisfaction with tooth appearance increased with increased dissatisfaction with teeth colour, feeling of poor tooth alignment, presence of fractured anterior teeth, and increased desire for orthodontic, crowns, and dentures treatments (*P* < .05). Dissatisfaction with tooth colour was associated with increased desire for tooth whitening and tooth coloured fillings (*P* < .05). *Conclusions*. Participants had high levels of dissatisfaction with tooth appearance and tooth colour. Dissatisfaction with tooth colour contributed to the increased dissatisfaction with tooth appearance. Dissatisfaction with tooth appearance, colour, alignment, and condition was significantly related to high desire for aesthetic treatments.

## 1. Introduction

Aesthetics of the orofacial region are very important aspects of human life. They might affect the quality of a patient's life. Among the most important goals of dental care is helping patients in their attempts to reach an acceptable level of satisfaction with their oral cavity and dentition [1]. Dentofacial conditions and status affect patients' appearance, performance, and function and shape patients' satisfaction with their dentition [2, 3]. Dental disease may influence the capacity to enjoy life, live comfortably, experience relationships, be successful in employment, and possess a positive self-image [4]. Variable oral situations as pain, speech, chewing ability, taste, and aesthetics affect various aspects of life quality as well as patient satisfaction with teeth [3, 5]. Dental professionals need an accurate perception of how patients feel about their teeth and the impact this has on their daily living. Consequently, dental care providers should be aware of the dental needs of patients, how patients feel about their teeth, and the impact this has on their satisfaction and daily living. Therefore, satisfaction and sociopsychological dimensions should be assessed whenever dental needs are assessed [3, 6].

Many factors affect aesthetics and might consequently affect satisfaction with appearance. These include presence of fillings and tooth colour, position, alignment, shape, and number [7–13].

Higher levels of satisfaction with appearance, better quality of life, and better psychological condition were associated with adequate dental aesthetics and dental treatments that improve dental aesthetics [14–20].

Aesthetic dental treatments including crowns, bleaching, orthodontic treatment, and tooth-coloured restorations are often desired by patients who seek better aesthetics [9, 13, 21, 22].

The aim of this study was to investigate participants' satisfaction with the appearance of their teeth and what are the desired treatments that patients seek to improve dental appearance.

## 2. Materials and Methods

In total, 220 university students (127 males and 93 females) were recruited into the study. Their age ranged from 18 to 27 years old (mean age = 21.4 ± 1.5 years). To be included in the study, recruited participants had to be 18 years old or older in order to understand and score the questionnaires, and had no medical disease or condition (including mental problems, cognitive disturbances, and psychological disorders) that might affect their ability to comprehend, understand, and/or score the questionnaires, and received no dental treatment for the last 6 months. The study was approved by the Research Committee, Faculty of dentistry, Al Jouf University, KSA. Each participant was given a brief explanation of the study, and an informed consent was obtained from each subject before being recruited into the study.

A structured questionnaire was used to assess patients' satisfaction with their appearance and what treatment they desire to improve their aesthetics (Table 1). The questionnaire was adopted from previous studies that found it valid and reliable [13]. It included items regarding sociodemographic data (such as age, gender, specialty of studies, and level of study) and items that tackle patient satisfaction with their teeth in general, tooth colour, tooth alignment, and tooth position. Also, the questionnaire included items that inquired about presence of caries, tooth-coloured fillings, and tooth fractures. Furthermore, the questionnaire included items that attempted to identify whether participants desire treatment to improve their appearance including orthodontics, bleaching, dental crowns, tooth-coloured fillings, and prosthetic rehabilitations and dentures. The questionnaire was modified for the purpose of this study by including a visual analogue scale for the participants to score the level of their satisfaction with their appearance in general. The VAS scale ranged from zero to ten where zero means the least satisfaction with appearance and ten means the maximum satisfaction with appearance.

The questionnaire was administered to the participants, and the process of completing the questionnaire was supervised by the investigator. Each participant was provided with a full explanation of the questionnaire as well as the method of scoring it.

The English version of the questionnaire was translated into Arabic language by three expert and fluent bilingual individuals and then backtranslated into English by another three individuals who were fluent in Arabic and English. Modifications to the questionnaire were made as necessary to ensure comprehension. Fifty undergraduate dental students at Al Jouf University were asked to score the English format of the questionnaire and then they were asked to score the translated Arabic version. The answers of the two formats of the questionnaire were compared using the *t*-test, and no statistical significant differences were found. The data obtained from the above procedure was not included in the main study. Then, the final version of the questionnaire was distributed to the participants in the main study sample.

Twenty participants answered the Arabic questionnaire twice with one-week interval. Reliability test was carried out on all questions using correlation coefficients. The correlation coefficients were high and ranged from 0.96 to 0.98.

### 2.1. Statistical Analysis

The data were analyzed using the SPSS computer software (Statistical Package for the Social Sciences, version 19.0, SPSS Inc., Chicago, IL, USA). First, simple descriptive frequency tests were carried out and processed. Then, the association between the variables was analyzed using the Pearson correlation test, while the ANOVA test was used to compare satisfied and dissatisfied patients. For all statistical analysis, the significance level was set at *P* ≤ 0.05.

## 3. Results

In total, 220 participants (127 males and 93 females) were recruited into the study. Their age ranged from 18 to 27 years old (mean age = 21.4 ± 1.5 years). Specialty of participants' field of study included medicine, dentistry, medical sciences and nursing, pharmacy, engineering, science, education, and preparatory year of study (*n* = 40, 23, 36, 22, 14, 24, 58, and 3 participants, resp.). Participants' study level ranged from first to fifth year.

Using VAS scale, the total satisfaction score of participants ranged from 0 to 10 (mean score = 6.8 ± 2.3). Ten (4.5%) participants were totally not satisfied with their appearance and scored 0 on the VAS scale, while 19 (8.6%) participants were totally satisfied with their appearance and scored 10 on the VAS scale, and 51 (23.2%) participants scored 5 or less on the VAS scale (Table 2).

Table 3 presents the distribution of the study sample by their answers to the questionnaire items. Half of the participants were dissatisfied with the appearance of their teeth, and 65.9% were dissatisfied with the colour of their teeth. Also, 25% of the participants felt that they had crowded teeth, 41.8% felt that their teeth were poorly aligned, and 17.7% felt that they had protruding teeth. Furthermore, 15.5% of the participants reported that they had caries, 16.4% had nonaesthetic fillings, and 23.2% had fractures in their anterior teeth (Table 3).

Correlations between the answers of the questionnaire items and each of age, gender, and specialty showed that younger participants had more fractures in anterior teeth (item 8, *P* = .014, *r* = −.165) and more dental caries in anterior teeth (item 6, *P* = .02, *r* = −.156). Also, males had less nonaesthetic fillings in anterior teeth (item 7, *P* = .012, *r* = .169) and had less desire to undergo denture treatment in order to improve the appearance of their teeth (*P* = .034, *r* = −.143). In addition, specialty of participants had significant relationship with the participants feel that their teeth were crowded (*P* = .017, *r* = .161), and participants desire to undergo dental crowning to improve the appearance of their teeth (*P* = .009, *r* = .175). Medicine and dentistry students had less feelings of having crowded teeth and less desire to undergo dental crowning treatment than education, science, and engineering students. Also, medicine and dentistry students had higher satisfaction scores on VAS scale than education, science, and engineering students (*P* = .046, *r* = −.134).

The lower satisfaction scores on VAS scale were associated with less satisfaction with appearance of teeth (items 1, *P* = .000, *r* = .262), feeling that teeth were poorly aligned (item 4, *P* = .002, *r* = −.212), having dental caries in anterior teeth (item 6, *P* = .000, *r* = −.250), having nonaesthetic fillings in anterior teeth (item 7, *P* = .000, *r* = −.242), and having fractures in anterior teeth (item 8, *P* = .001, *r* = −.221). Also, lower satisfaction scores on VAS scale were associated with higher participants' desire to improve the appearance of their teeth by undergoing the following treatments: orthodontic treatment (*P* = .003, *r* = −.202), tooth whitening (*P* = .035, *r* = −.143), dental crowning (*P* = .004, *r* = −.193), tooth-coloured fillings (*P* = .007, *r* = −.183), and denture prosthesis (*P* = .012, *r* = −.169).

Also, participants' satisfaction with appearance of teeth (item 1) was associated with more satisfaction with tooth colour (items 2, *P* = .000, *r* = −.355), less feelings that teeth were poorly aligned (item 4, *P* = .000, *r* = −.258), and having less fractures in anterior teeth (item 8, *P* = .038, *r* = −.140). Also, participants' satisfaction with appearance of teeth (item 1) was associated with less desire to improve the appearance of their teeth by undergoing the following treatments: orthodontic treatment (*P* = .007, *r* = −.182), dental crowning (*P* = .005, *r* = −.188), and denture prosthesis (*P* = .038, *r* = −.140).

Participants' satisfaction with the colour of their teeth (item 2) was associated with less desire to undergo tooth-whitening (*P* = .000, *r* = −.334) and tooth-coloured fillings (*P* = .039, *r* = −.139) to improve the appearance of their teeth. Participants' feeling of having crowded teeth (item 3) was associated with more desire to undergo orthodontic treatment (*P* = .000, *r* = .242) and denture prosthesis (*P* = .000, *r* = .280) to improve the appearance of their teeth. Participants' feeling that teeth were poorly aligned (item 4) was associated with more desire to undergo orthodontic treatment (*P* = .000, *r* = .393), dental crowning (*P* = .018, *r* = .160), and denture prosthesis (*P* = .031, *r* = .146) to improve the appearance of their teeth. Participants' feeling that their teeth were protruding (item 5) was associated with more desire to undergo orthodontic treatment (*P* = .000, *r* = .305), dental crowning (*P* = .019, *r* = .158), and denture prosthesis (*P* = .003, *r* = .196) to improve the appearance of their teeth. Having dental caries (item 6) was associated with more desire to undergo tooth-coloured filling (*P* = .039, *r* = .139) and denture prosthesis (*P* = .007, *r* = .182) to improve the appearance of their teeth. Having nonaesthetic fillings in anterior teeth (item 7) was associated with more desire to undergo orthodontic treatment (*P* = .021, *r* = .156), tooth whitening (*P* = .024, *r* = .152), dental crowning (*P* = .005, *r* = .188), and tooth-coloured filling (*P* = .000, *r* = .247) to improve the appearance of their teeth. Having fractures in anterior teeth (item 8) was associated with more desire to undergo orthodontic treatment (*P* = .006, *r* = .185), dental crowning (*P* = .009, *r* = .175), and denture prosthesis (*P* = .006, *r* = .183) to improve the appearance of their teeth.

Table 4 presents distribution of satisfied (*n* = 110) and dissatisfied (*n* = 110) participants by the mean VAS scale score of satisfaction and participants' sociodemographic factors. Participants who answered yes for the item “Are you satisfied with the general appearance of your teeth?” scored significantly higher values on VAS scale than those whose answer was no (*P* = .000) (Table 4). However, no significant differences were found between the two groups in terms of gender, age, and specialty (*P* > .05) (Table 4).

Table 5 presents the distribution of satisfied (*n* = 110) and dissatisfied (*n* = 110) participants by their answers to the questionnaire items. Participants who answered yes for the item “Are you satisfied with the general appearance of your teeth?” were significantly more satisfied with the colour of their teeth, felt that their teeth were better aligned, and had less fractures in their front teeth than those whose answer was no (*P* = .000, .000, and .038, resp.) (Table 5). However, no significant differences were found between the two groups in terms of feeling of tooth crowding, feeling of tooth protrusion, having caries in front teeth, and having nonaesthetic fillings in their front teeth (*P* > .05) (Table 5).

## 4. Discussion

This study investigated satisfaction with appearance, dental appearance, and desire for treatment to improve dental appearance. Perception of dental appearance differs between individuals and populations [23]. In this study, 50% of participants were satisfied with the appearance of their teeth. Previous studies in different populations showed different levels of satisfaction among the studied samples, for example, 47.2% in Malaysia [13], 57.3% in Turkey [11], 65% in Palestine [20], 65.5% in Jordan [15], and 76% in UK [24]. This could be attributed to the use of different measures to evaluate satisfaction, cultural factors, religion, and racial factors as well as to that dental appearance is affected by individual characteristics, compliance, or unrealistic expectations [7, 13, 15, 21].

Nearly 66% of the participants were dissatisfied with the colour of their teeth. Other studies reported higher levels of satisfaction with tooth colour [9, 11, 13]. This could be attributed to the difference in sample size and used measures to evaluate satisfaction, psychological factors, and religious and sociocultural factors.

Also, satisfaction with tooth colour was significantly related to satisfaction with dental appearance. This finding supports the idea that satisfaction with tooth colour strongly impacts on satisfaction with dental appearance [9, 13]. Furthermore, tooth whitening was found to be the most desired treatment by participants in order to improve tooth appearance, and this further supports the impact of tooth colour on satisfaction with dental appearance. This finding concurs the results of previous studies [9, 13].

Satisfaction with dental appearance was significantly related to the feeling of presence of poor tooth alignment and presence of fractured anterior teeth. This could be due to that poor tooth alignment and anterior tooth fractures change the appearance of teeth causing them to be less attractive. This was supported by the finding that satisfaction with dental appearance had significant relation to the desire for orthodontic, dental crowning, and denture treatments in order to improve the appearance of teeth. This concurs the results of previous studies [9] but contrasts with others [13].

Age had no relation to satisfaction with appearance of teeth. This concurs the results of previous studies [13, 15, 21, 25]. However, it disagrees with the results of previous studies that found more satisfaction with appearance among older subjects [11, 20, 24].

Again, this could be attributed to the use of different measures to evaluate satisfaction and also to that dental appearance is affected by individual characteristics as well as cultural, religious, or racial factors [7, 13, 15, 21].

Gender had no relation to satisfaction with appearance of teeth and tooth colour. This is similar to the findings reported by previous studies [11, 15, 20, 21]. However, the results disagree with the findings of previous studies that found less satisfaction with dental appearance among females [9, 13, 23].

Different samples, different measuring techniques, individual characteristic, and psychology, as well as cultural, religious, and racial back grounds might explain this controversy regarding the relationship between gender and satisfaction with dental appearance and tooth colour.

In this study, satisfaction with general appearance (identified by VAS scale) had a significant relation to satisfaction with teeth. This concurs the idea that satisfaction with teeth and tooth colour contributes to the overall satisfaction with appearance [10, 15, 20]. This was further supported by the findings of this study where satisfaction with general appearance was found to have a significant relation with participants' desire for all types of treatments that could improve the appearance of teeth. In contrast, other studies found no relation between satisfaction with dental appearance and satisfaction with general appearance [21, 26]. Psychological factors and different samples might explain the controversy in this regard.

Cultural, social, psychological, economic, or religious factors in different populations might affect satisfaction with the appearance of teeth. Further studies are required to identify the potential effects of such factors in this regard.

## 5. Conclusions

High levels of dissatisfaction with tooth appearance and tooth colour were reported in this study. Higher levels of dissatisfaction with tooth colour contributed to the increased dissatisfaction with appearance of teeth. Specialty, gender, and age were not related to satisfaction. Dissatisfaction with tooth appearance, colour, alignment, and condition were significantly related to the high desire for treatments that improve dental aesthetics.

## Figures and Tables

**Table 1:** The used questionnaire in the study.

Satisfaction with appearance and the desired treatment to improve aesthetics		
Name:	Age:	Gender:
Speciality:	Study level:	

Please answer the following questions.		
(1) Are you satisfied with the general appearance of your teeth?	□ Yes	□ No
(2) Are you satisfied with your tooth color?	□ Yes	□ No
(3) Do you feel your teeth are crowded?	□ Yes	□ No
(4) Do you feel your teeth are poorly aligned?	□ Yes	□ No
(5) Do you feel your teeth are protruding?	□Yes	□ No
(6) Do you have dental caries in your front teeth?	□ Yes	□ No
(7) Do you have non-aesthetic fillings in your front teeth?	□ Yes	□ No
(8) Do you have fractures in your front teeth?	□ Yes	□ No
(9) Do you wish to undergo these treatments to improve the appearance of your teeth?		
(a) Orthodontic treatment to realign teeth	□ Yes	□ No
(b) Tooth whitening	□ Yes	□ No
(c) Dental crowns	□ Yes	□ No
(d) Tooth-coloured fillings	□ Yes	□ No
(e) Dentures	□ Yes	□ No
(10) Please give a score of your satisfaction with your appearance in general out of 10 where 10 is maximum satisfaction and zero is not satisfied at all: (please write the score here)…		

**Table 2:** Distribution of satisfaction score among the study population (*N* = 220).

Satisfaction score on VAS scale	Frequency of participants	Percentage of participants
0	10	4.5
1	1	.5
2	2	.9
4	11	5.0
5	27	12.3
6	28	12.7
7	48	21.8
8	50	22.7
9	24	10.9
10	19	8.6

Total	220	100.0

**Table 3:** Distribution of the study sample by their answers to the questionnaire items.

Item	Participant's answers (*n* = 220)	
	Yes (%)	No (%)
(1) Are you satisfied with the general appearance of your teeth?	110 (50)	110 (50)
(2) Are you satisfied with your tooth color?	75 (34.1)	145 (65.9)
(3) Do you feel your teeth are crowded?	55 (25)	165 (75)
(4) Do you feel your teeth are poorly aligned?	92 (41.8)	128 (58.2)
(5) Do you feel your teeth are protruding?	39 (17.7)	181 (82.3)
(6) Do you have dental caries in your front teeth?	34 (15.5)	186 (84.5)
(7) Do you have non-aesthetic fillings in your front teeth?	36 (16.4)	184 (83.6)
(8) Do you have fractures in your front teeth?	51 (23.2)	169 (76.8)
(9) Do you wish to undergo these treatments to improve the appearance of your teeth?		
(a) Orthodontic treatment to realign teeth	114 (51.8)	106 (48.2)
(b) Tooth whitening	178 (80.9)	42 (19.1)
(c) Dental crowns	67 (30.5)	153 (69.5)
(d) Tooth coloured	76 (34.5)	144 (65.5)
(e) Dentures	51 (23.2)	169 (76.8)

**Table 4:** Distribution of satisfied (*n* = 110) and dissatisfied (*n* = 110) participants by mean VAS scale score of satisfaction and participants' sociodemographic factors.

Item	Satisfied group, *n* = 110 (%)	Dissatisfied group, *n* = 110 (%)	*P* value using ANOVA test*, (F)
Mean VAS scale score (SD)	7.4 (2.2)	6.2 (2.2)	.000 (16.078)
Gender			
Male	60	67	.342 (.908)
Female	50	43	
Mean age (SD)	21.5 (1.5)	21.2 (1.4)	.136 (2.237)
Specialty			
Medicine	19 (17.3)	21 (19.1)	.077 (3.156)
Dentistry	14 (12.7)	9 (8.2)	
Medical sciences and nursing	24 (21.8)	12 (10.9)	
Pharmacy	10 (9.1)	12 (10.9)	
Engineering	8 (7.3)	6 (5.5)	
Science	7 (6.4)	17 (15.5)	
Education	27 (24.5)	31 (28.2)	
Preparatory year of study	1 (.9)	2 (1.8)	
Level of study			
1	4 (3.6)	8 (7.3)	.002 (10.164)
2	37 (33.6)	41 (37.3)	
3	23 (20.9)	35 (31.8)	
4	29 (26.4)	23 (20.9)	
5	7 (6.4)	2 (1.8)	
6	9 (8.2)	1 (.9)	
7	1 (.9)	0 (0%)	

*Degree of freedom (df) = 1, F: F-Statistic, VAS: visual analogue scale, SD: standard deviation.

**Table 5:** Distribution of satisfied (*n* = 110) and dissatisfied (*n* = 110) participants by mean VAS score of satisfaction, answers to the questionnaire items, and their socio-demographic factors.

Item	Satisfied group, *n* = 110 (%)	Dissatisfied group, *n* = 110 (%)	*P* value using ANOVA test, (F)
(Q2) Are you satisfied with your tooth color?			
No	54 (49.1)	91 (82.7)	.000 (31.395)
Yes	56 (50.9)	19 (17.3)	
(Q3) Do you feel your teeth are crowded?			
No	85 (77.3)	80 (72.7)	.439 (.602)
Yes	25 (22.7)	30 (27.3)	
(Q4) Do you feel your teeth are poorly aligned?			
No	78 (70.9)	50 (45.5)	.000 (15.549)
Yes	32 (29.1)	60 (54.5)	
(Q5) Do you feel your teeth are protruding?			
No	94 (85.5)	87 (79.1)	.218 (1.524)
Yes	16 (14.5)	23 (20.9)	
(Q6) Do you have dental caries in your front teeth?			
No	97 (88.2)	89 (80.9)	.137 (2.229)
Yes	13 (11.8)	21 (19.1)	
(Q7) Do you have nonaesthetic fillings in your front teeth?			
No	94 (85.5)	90 (81.8)	.468 (.528)
Yes	16 (14.5)	20 (18.2)	
(Q8) Do you have fractures in your front teeth?			
No	91 (82.7)	78 (70.9)	.038 (4.360)
Yes	19 (17.3)	32 (29.1)	
(Q9) Do you wish to undergo these treatments to improve the appearance of your teeth?			
(a) Orthodontic treatment to realign teeth			
No	63 (57.3)	43 (39.1)	.007 (7.463)
Yes	47 (42.7)	67 (60.9)	
(b) Tooth whitening			
No	24 (21.8)	18 (16.4)	.306 (1.055)
Yes	86 (78.2)	92 (83.6)	
(c) Dental crowns			
No	86 (78.2)	67 (60.9)	.005 (7.957)
Yes	24 (21.8)	43 (39.1)	
(d) Tooth coloured			
No	75 (68.2)	69 (62.7)	.397 (.719)
Yes	35 (31.8)	41 (37.3)	
(e) Dentures			
No	91 (82.7)	78 (70.9)	.038 (4.360)
Yes	19 (17.3)	32 (29.1)	

*Degree of freedom (df) = 1, F: F-Statistic, VAS: visual analogue scale, SD: standard deviation.
